# T204. NOVEL VIRTUAL REALITY SOCIAL SKILLS TRAINING FOR INDIVIDUALS WITH SCHIZOPHRENIA

**DOI:** 10.1093/schbul/sby016.480

**Published:** 2018-04-01

**Authors:** Lénie Torregrossa, Laura Hieber Adery, Megan Ichinose, Heathman Nichols, Alena Gizdić, Joshua Wade, Dayi BIan, Eric Granholm, Nilanjan Sarkar, Sohee Park

**Affiliations:** 1 Vanderbilt University; 2 University of California, San Diego

## Abstract

**Background:**

Social impairment is a core feature of schizophrenia presenting a major barrier to recovery. Although antipsychotic medications can reduce psychotic symptoms, social functioning often remains poor, contributing to the financial burden of schizophrenia. Validated behavioral interventions, such as Social Skills Training (Liberman & Martin, 1988), target a broad range of social domains by practicing pragmatic living skills. But they yield only modest effect sizes for social outcome (Pfammatter et al, 2006). Conventional social interventions present further limitations including: time and effort required from patients and therapists, low adherence, lack of personalization, and low generalizability. Importantly, most people with mental illness do not currently have access to social interventions. The aim of this study was to design and implement an effective, high-compliance virtual reality (VR) social skills training game for people with schizophrenia.

**Methods:**

The advantages of the VR environment include flexibility, controllability, extensive repertoire of stimuli, low-burden, low-cost and safety (Strickland, 1997). The goal of the training game was to support social attention to improve social skills learning. We trained social skills by exercising problem-solving in naturalistic scenarios: the grocery store, a bus stop, and a cafeteria. Subjects moved through variable steps in a social “mission” to obtain personal information through conversations with avatars. Each mission began with the participant fixating on the avatar. Subjects then had to decide which avatar to approach and choose an appropriate response to the avatar’s prompts. If they chose an incorrect response, oral feedback was provided on why this response was not the most effective, and instructed them to try again. This occurred until the participant identified the most appropriate response, thus completing the mission and getting access to the next level of difficulty. Each training session concluded after completion of 12 total conversation missions.

Eighteen individuals with schizophrenia (SZ) and twenty demographically matched controls (CO) participated in this study. At baseline, SZ and CO completed pre-training assessments. The CO group did not undergo VR training but participated in behavioral assessments so that we could compare SZ performance to normative data. SZ participated in the VR training twice a week for 5 weeks (10 sessions). After the 10th session, we re-examined social functioning, cognitive functions, and symptoms. SZ also completed a satisfaction survey upon training completion.

**Results:**

Of the eighteen SZ participants enrolled in the study, sixteen completed the 10 sessions of training, yielding a retention rate of 89%. 80% of SZ subjects reported being “extremely satisfied” with the training. None reported not being satisfied. 93.3% rated the training as “acceptable” and the effort required to attend the study as “easy.” Regarding outcome, negative symptoms significantly decreased from pre-training to post-training. Performance on BLERT and CogState Social Emotional Cognitive Task significantly improved. The average time taken to complete a mission was significantly lower in the last session compared to the first, showing that participants became increasingly better at efficiently solving these social missions.

**Discussion:**

These results show evidence for VR training as an acceptable and feasible intervention improving social functioning in SZ. Future work will test the adaptive social VR training against an active control condition in a pilot randomized controlled trial to evaluate the relative efficacy of the VR training on enhancing social attention and associated neural circuitry.

